# pH Dependent Antimicrobial Peptides and Proteins, Their Mechanisms of Action and Potential as Therapeutic Agents

**DOI:** 10.3390/ph9040067

**Published:** 2016-11-01

**Authors:** Erum Malik, Sarah R. Dennison, Frederick Harris, David A. Phoenix

**Affiliations:** 1 School of Forensic and Applied Sciences, University of Central Lancashire, Preston PR1 2HE, UK; EErum@uclan.ac.uk (E.M.); Fharris1@uclan.ac.uk (F.H.); 2 School of Pharmacy and Biological Sciences, University of Central Lancashire, Preston PR1 2HE, UK; srdennison1@uclan.ac.uk; 3 Office of the Vice Chancellor, London South Bank University, 103 Borough Road, London SE1 0AA, UK

**Keywords:** antimicrobial peptides and proteins, pH dependent antimicrobial activity, invertebrates, vertebrates

## Abstract

Antimicrobial peptides (AMPs) are potent antibiotics of the innate immune system that have been extensively investigated as a potential solution to the global problem of infectious diseases caused by pathogenic microbes. A group of AMPs that are increasingly being reported are those that utilise pH dependent antimicrobial mechanisms, and here we review research into this area. This review shows that these antimicrobial molecules are produced by a diverse spectrum of creatures, including vertebrates and invertebrates, and are primarily cationic, although a number of anionic examples are known. Some of these molecules exhibit high pH optima for their antimicrobial activity but in most cases, these AMPs show activity against microbes that present low pH optima, which reflects the acidic pH generally found at their sites of action, particularly the skin. The modes of action used by these molecules are based on a number of major structure/function relationships, which include metal ion binding, changes to net charge and conformational plasticity, and primarily involve the protonation of histidine, aspartic acid and glutamic acid residues at low pH. The pH dependent activity of pore forming antimicrobial proteins involves mechanisms that generally differ fundamentally to those used by pH dependent AMPs, which can be described by the carpet, toroidal pore and barrel-stave pore models of membrane interaction. A number of pH dependent AMPs and antimicrobial proteins have been developed for medical purposes and have successfully completed clinical trials, including kappacins, LL-37, histatins and lactoferrin, along with a number of their derivatives. Major examples of the therapeutic application of these antimicrobial molecules include wound healing as well as the treatment of multiple cancers and infections due to viruses, bacteria and fungi. In general, these applications involve topical administration, such as the use of mouth washes, cream formulations and hydrogel delivery systems. Nonetheless, many pH dependent AMPs and antimicrobial proteins have yet to be fully characterized and these molecules, as a whole, represent an untapped source of novel biologically active agents that could aid fulfillment of the urgent need for alternatives to conventional antibiotics, helping to avert a return to the pre-antibiotic era.

## 1. Introduction

A multiplicity of synergistic factors, including diminished pharmaceutical investment, clinical over-prescription and misuse by the food industry has led to the increasing occurrence of microbial pathogens with multiple drug resistance (MDR) and rendered infectious diseases the leading cause of global mortality [1]. This bleak situation led the World Health Organization (WHO) to recently predict that the uncurbed rise of MDR pathogens could see conditions in the 21st Century return to those of the pre-antibiotic era when no antimicrobials were available for the treatment of many common diseases [2]. In response, a major analysis by the WHO and a report from the O’Neill review, sponsored by the UK Government, have concluded that the problem of antimicrobial drug resistance can only be fully addressed by a coordinated global approach that operates through a number of major interventions (Table 1) [3]. In particular, intervention six (Table 1) proposed the urgent development of novel products and strategies that could provide alternatives to conventional antibiotics, which has generated intensive research into antimicrobial design [4]. Examples of this research range from revisiting old anti-infective strategies, such as phage therapy, which was popular in Eastern European countries in the early 20th Century [5], to recently reported antimicrobial strategies, such as the development of compounds whose antibiotic activity can be regulated by light and sound [4,6,7,8]. One particularly promising approach proposed by O’Neill [3] was the therapeutic development of antimicrobial peptides (AMPs), which are potent antibiotics of the innate immune system [9,10]. The activity of these peptides against microbes involves relatively non-specific modes of action at multiple sites with the result that microbial resistance to AMPs has a low incidence and is generally due to inherent rather than adaptive mechanisms [11]. Based on these observations, the generally held view is that microbial resistance to AMPs is unlikely to approach those of conventional antibiotics, endowing these peptides with a major medical advantage [10], and currently, a number of AMPs are in clinical trials (Table 2).

In order to develop AMPs as medically relevant anti-infective agents, there have been numerous investigations into their antimicrobial mechanisms, which to date has shown that membrane interaction is a requirement for virtually all of these mechanisms [9,10,16,17]. These investigations have also shown that there are a number of major drivers in the membrane interactions of AMPs of which the most important are charge, hydrophobicity and amphiphilicity [9,18,19]. The vast majority of AMPs are cationic to help facilitate the targeting of microbes through direct electrostatic interaction with anionic components of their membranes [19,20]. Nearly all AMPs are also amphiphilic, which generates hydrophobic surfaces that are able to drive the partitioning of these peptides into microbial membranes and hydrophilic surfaces that are able to stabilize these hydrophobic interactions via electrostatic associations with the head group regions of these membranes [21,22]. Based on these investigations, a variety of models have been proposed to describe the antimicrobial action of AMPs with those most frequently reported appearing to be variants of the barrel-stave pore and carpet type mechanisms, which involve membrane disruption via discrete channel formation and non-specific solubilization respectively [23].

There have been many advances in understanding the mode of action used by AMPs but, although a number of earlier studies showed that pH can modulate the antimicrobial activity of these peptides, no major review of this area of research appears to have been presented in the literature [24,25,26,27,28]. However, it is now becoming increasingly clear that pH is a major driver in the membrane interactions and biological activity of not only many AMPs but also a number of antimicrobial proteins produced by eukaryotes (Table 3). To provide an update on these antimicrobial molecules, here, we present an overview of recent progress in the understanding of their modes of action along with the development of their therapeutic and biotechnological potential.

## 2. An Overview of pH Dependent Peptides and Proteins with Antimicrobial Activity

In the 1980s and 1990s, a series of seminal studies, including work on the African clawed frog, *Xenopus laevis*, and a number of mammals, led to what many take to be the first major description of eukaryotic AMPs such as magainins, defensins and surfactant-associated anionic peptides (SAAPs) [90]. However, in 1956, phagocytin from humans, rabbits, horses and guinea pigs was reported to exhibit non-membranolytic activity against a range of Gram-positive and Gram-negative bacteria that was enhanced by low pH [24,25]. The peptide was not characterized or further investigated and today, it is not even known as to whether phagocytin was rediscovered later and given an alternative name [91]. However, it would appear to be a matter of historical fact that what was most likely the first AMP to be reported from eukaryotes showed a pH dependent mode of action [24,25]. Since these earlier studies, it is now known that these peptides are produced by virtually all multicellular organisms [92,93] and that an increasing number of these molecules possess pH-dependent activity (Table 3).

### 2.1. Fish

Gaduscidin-1 (gad-1) and gaduscidin-2 (gad-2) were AMPs identified in the Atlantic cod, *Gadus morhua*, and shown to be highly, constitutively expressed in immune-relevant tissues [94,95]. Low pH was found to enhance the activity of both peptides against *Escherichia coli*, which appeared to involve membrane interaction, but interestingly, although gad-1 and gad-2 were predominantly α-helical at neutral pH, acid conditions led to a large decrease in the levels of α-helicity possessed by these peptides [29]. These results contrast with most α-helical AMPs where an enhanced capacity for membranolysis and antimicrobial activity is generally associated with increased levels of this secondary structure [96,97]. It was proposed that gad-1 and gad-2 each possessed a structural plasticity, which facilitated an appropriate balance between amphiphilic and mixed hydrophobic/hydrophilic structural features that promoted maximal levels of membrane interaction and antibacterial activity [29]. Gad-1 and gad-2 were found to be histidine rich AMPs and the enhanced capacity of these peptides for membranolysis and antimicrobial activity at low pH appeared to involve these residues [29]. It is well established that histidine (pKa 6.5) is uncharged at physiological pH but fully positively charged at low pH, thereby enhancing the potential of AMPs for interaction with anionic membranes under acid conditions [46,98]. However, there also appeared to be a complex relationship between the level of histidine residues possessed by these AMPs and their pH dependent capacity for membrane interaction [29,30]. In response, molecular dynamic simulation studies were undertaken and predicted that the number of sequential histidine pairs contained by gad-1 and gad-2 were important to their ability for membrane disruption [30]. These AMPs possess one and two of these histidine pairs respectively [94] and the N-terminal regions of both peptides, which included this motif, were preferentially located proximal to membrane channels with which gad-1 and gad-2 were associated [30]. Based on the topology of the peptide–lipid interactions mediating the formation of these channels, it was suggested that the antimicrobial action of gad-1 and gad-2 may involve the use of a disordered toroidal pore type mechanism of membrane disruption [30,99,100].

### 2.2. Amphibians

**Figure 1:**
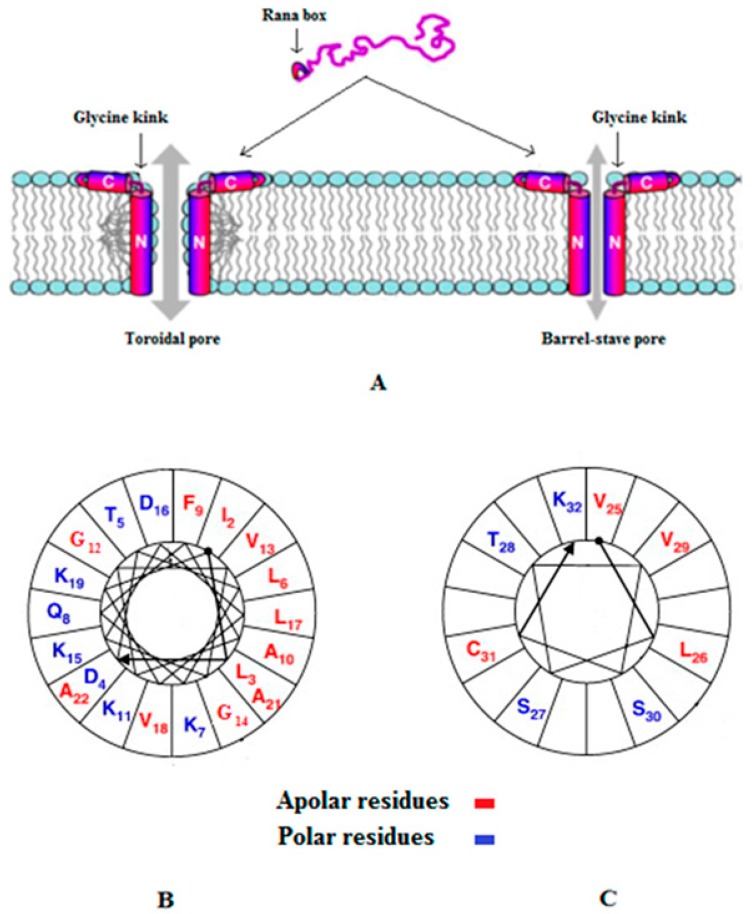
Models for the membrane pore formation by E2EM. Figure 1 was revised from [115] and Figure 1A shows models for pore formation by E2EM, which are the toroidal pore and barrel stave mechanisms (Table 1) and are the best supported experimentally. In both models, the N-terminal 23 residues of the peptide spans the bilayer and a glycine kink orientates the 7 residue, C-terminal Rana box region of E2EM to lie parallel to the membrane surface. In this orientation, the Rana box region of the peptide, which is a cystein stabilized macrocyclic structure, interacts with the lipid head-group region of the membrane and stabilizes pore formation by E2EM [115]. The major difference between these models is that in the toroidal pore mechanism, the membrane leaflets deform to allow the lipid head-group region to remain in contact with the hydrophilic face of the E2EM membrane spanning region, which is not observed in the barrel stave mechanism [23]. For clarity, two monomers of E2EM are shown in the schematic pore above but oligomers formed by between five and ten peptide molecules have been proposed [115,120]. Similar models of membrane interaction appear to apply to the linear reduced form of the peptide [115], which is represented in our studies as E2EM-lin. Figure 1B,C show two-dimensional axial projections [126] for the membrane spanning region and Rana box domain of E2EM, respectively, that are involved in pore formation by the peptide. In both cases, these segments for amphipilic α-helices with wide hydrophobic faces that our data suggest would be maximized by alkaline pH, thereby promoting the potential for the mutual interaction of E2EM monomers and the formation of multimeric species involved in pore formation.

Chensinin-1 is a histidine rich peptide produced by the frog, *Rana chensinensis* and recent studies showed that low pH enhanced the positive charge of the peptide [31] and thereby, its ability to kill Gram-positive bacteria, such as *Bacillus cereus* [32]. This antibacterial activity appeared to involve the adoption of an extended structure, similar to that of other AMPs that are rich in specific residues [101,102], which induced lysis of the *B. cereus* membrane [32]. Interestingly, the peptide showed no activity against Gram-negative bacteria [32,103], which appeared to involve high affinity binding between chensinin-1 and lipopolysaccharide (LPS) in the outer membrane of these bacteria [103]. Maximin H5 from the toad, *Bombina maxima*, was also recently found to be ineffective against Gram-negative bacteria due to high affinity binding to phosphatidylethanolamine (PE) in the cytoplasmic membrane (CM) of these organisms [104,105]. Similar PE mediated mechanisms have been proposed to mediate the resistance of microbes to other AMPs [104,105], supporting the growing view that receptors could play a variety of roles in the biological activities of these peptides [23,98,106,107,108]. Esculentin-2EM (E2EM, previously gaegurin 4) is an α-helical peptide isolated from the frog, *Glandirana emeljanovi* (formerly *Rana rugosa*) [109,110], that is able to kill protozoa, fungi, Gram-positive bacteria and Gram-negative bacteria [110,111,112]. E2EM possesses a C-terminal cyclic region stabilized by a disulphide bond (Rana box) that is conserved across many ranid AMPs and helps stabilize pore formation by the peptide thereby promoting its antimicrobial action (Figure 1) [109,110,112,113,114,115]. Several models have been proposed to represent pore formation by E2EM and the best, supported by experimental evidence, appear to be the toroidal pore and barrel stave mechanisms (Figure 1). Here, we present data showing that the linear reduced form of E2EM (E2EM-lin) possesses antimicrobial activity consistent with recent studies showing that the reduction of cysteine-stabilized AMPs to generate peptides with novel mechanisms of antimicrobial activity may form part of some innate immune systems [116,117]. Our results showed that E2EM-lin was active against both Gram-positive and Gram-negative bacteria and appeared to exhibit pH dependent antimicrobial activity, which parallels the molluscan cysteine stabilized AMPs, myticins, whose reduced forms were described above to show pH dependent antibacterial and antiviral action [58,59]. It was found that under the low pH conditions associated with the skin of frogs [118], E2EM-lin had a general ability to induce the lysis of both anionic and zwitterionic membranes, which was enhanced at higher pH (Table 4). These data clearly suggest that the C-terminal disulphide bond in the E2EM Rana box region does not play a major role in its ability for membrane pore formation (Figure 1), which supports earlier work [114,115]. In the case of Dimyristoyl-phosphatidylglycerol (DMPG), which is a key component of membranes within Gram-positive bacteria [119], E2EM-lin induced relatively low levels of membrane lysis at acid pH (<25%). However, a shift to alkaline pH led to a large increase in the lytic activity of the peptide to circa 95%, which was accompanied by a correspondingly large increase in its α-helical content of circa 25% to give levels approaching 75% (Table 4). Previous studies have shown that E2EM-lin and E2EM adopt highly similar α-helical structures in membranes and undergo oligomerization to form pores [115,120]. Based on these data, we speculate that high pH may enhance the ability of E2EM-lin to lyse DMPG membranes by increasing the potential of the peptide for pore formation through an increased capacity for self-association. The segments of E2EM-lin involved in pore formation are strongly amphiphilic α-helices with wide hydrophobic faces that would be maximized by alkaline pH, promoting the potential for the mutual interactions involved in the formation of multimeric species (Figure 1). In the case of DMPE, which is often taken to represent the membranes of Gram-negative bacteria [119], E2EM-lin induced high levels of membrane lysis at acid pH (60%). A move to alkaline pH led to a relatively low increase in the lytic activity of the peptide, which was around 25% and was accompanied by a decrease in its α-helical content of 20% to give levels of circa 30% (Table 4). These data would seem to indicate that E2EM-lin uses a different mechanism of lysis in the case of DMPE membranes and it has previously been suggested that the peptide may adopt a number of lipid interactive forms [115]. The functional significance of our data is not fully clear but given the very high levels of membrane lysis induced by E2EM-lin at higher pH for each lipid investigated, biological relevance is suggested, which forms the basis of ongoing investigations.

In addition to cationic AMPS, anionic AMPs with pH dependent activity have also been reported for amphibians, such as dermaseptin PD-3-7, which was isolated from the frog, *Pachymedusa dacnicolor* [121]. In aqueous solution, the peptide showed an inherent propensity to adopt an extended conformation and self-assemble into amyloid fibrils in a reversible pH-controlled manner [33]. At low pH, dermaseptin PD-3-7 existed as amyloid-like β-sheet aggregates but at higher pH underwent morphological changes, which led to the formation of metastable amorphous aggregates in a manner that appeared to be mediated by deprotonation of the aspartic acid residues (pKa 3.9) and C-terminal carboxyl groups possessed by the peptide. These amorphous aggregates induced damage to cells of the insect, *Spodoptera frugiperda*, by an unidentified mechanism but showed no activity against *E. coli* and Bacillus subtilis [33]. Based on these observations, it was suggested that amyloid formation by dermaseptin PD-3-7 may act as a storage facility for the peptide similar to the depository function proposed for the amyloidogenesis of pituitary peptide hormones [122]. Triggered by an increase in pH, this storage facility would release a pre-formed, cytotoxic agent that contributed to the natural defense strategy of the host amphibian [33]. However, it is worthy of note that the peptide was only tested for activity against a small number of bacteria [33] and it is generally accepted that AMPs are promiscuous in their antimicrobial mechanisms [90,123] with amyloid-mediated antibacterial mechanisms increasingly being reported [23,124]. Interestingly, more recent studies on dermaseptin PD-3-7 have shown that stereochemical modification of the peptide’s second residue to form the diastereomer [d-Leu2] strongly influenced the pH-triggered, morphological changes involved in amyloid formation by dermaseptin PD-3-7, inducing a fundamental change in its superstructural organization that was related to differences between the conformational propensities of these epimers [125]. It was proposed by these latter authors that epimers of PD-3-7 may play a role as anionic AMPs, or defense molecules, in the innate immune system of *P. dacnicolor* [125] and a similar proposal has been made for the production of epimeric AMPs by other frogs and toads [9].

### 2.3. Humans and Other Mammals

**Figure 2:**
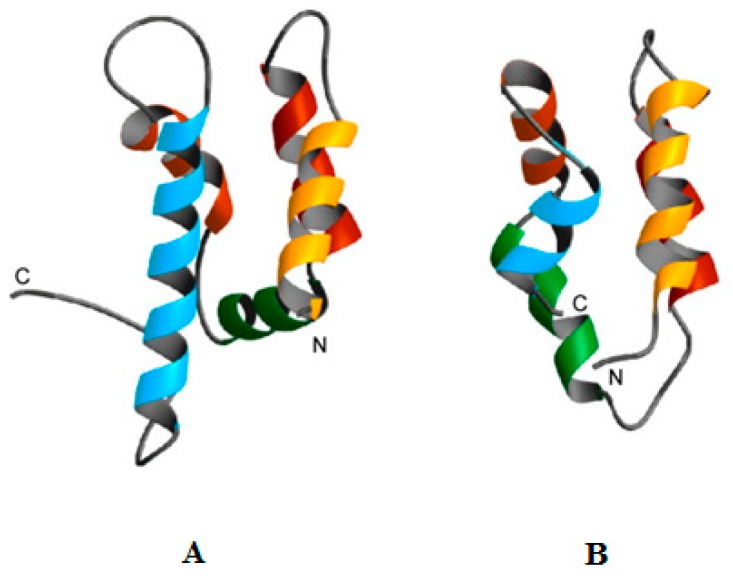
Similarities between the structures of psoriasin and amoebapore A. Figure 2 was revised from [35] and shows human psoriasin (**A**) and amoebapore A from the protozoa, *Entamoeba histolytica* (**B**). It can be clearly seen that these peptides show structural similarities and both have been shown to possess pH dependent mechanisms of antimicrobial activity that is enhanced by acid conditions [34,35,36,79,80,81,83]. In particular, psoriasin possesses a histidine residue in its C-terminal region [127] similarly to amoebapore A [82] and based on these similarities, it can be speculated that the enhanced antibacterial action of psoriasin at low pH may involve a histidine mediated increased ability for pore formation and oligomerisation.

Psoriasin (S100A7) is a human, cystein stabilized α-helical protein [127] of the S100 family of signaling proteins [128,129,130] that is known to have a role in the antimicrobial defense of the skin, including serving as a multifunctional modulator of neutrophil activation [131,132]. The protein was investigated for antimicrobial activity and shown to kill *E. coli* using pH independent mechanisms that were primarily due to the depletion of Zn^2+^ [34,35] and more recently, the protein was identified in vaginal fluid, appearing to help protect the female genital tract from infection [36]. Psoriasin was also found to kill *Baciillus megaterium* but via the use of two different modes of action, which involved Zn^2+^ depletion at neutral pH but membrane pore formation and oligomerisation of the protein at low pH. This pore forming mechanism was not further investigated but evidence suggested that it was likely to show some similarities to a barrel-stave pore type mode of action [34,35]. It is interesting to note that psoriasin exhibits a number of structural and functional similarities to amoebapore A (Figure 2) [35,133], which is a pH dependent antimicrobial protein from the protozoa, Entamoeba histolytica that is discussed below [81]. In particular, psoriasin possesses a histidine residue in its C-terminal region [127] similar to amoebapore A [82] and based on these similarities, it can be speculated that the enhanced action of psoriasin against B. megaterium at low pH may involve a histidine mediated increased ability for pore formation and oligomerisation. β-microseminoprotein (MSP), also named as PSP-94, is a human protein that is believed to have a protective role in prostate carcinogenesis due to its ability to suppress the growth of tumors although more recent studies have suggested that MSP may protect against prostate cancer by inhibiting fungal infection in this genital region [134,135]. This suggestion was primarily based on recent work, which showed that the acid conditions of the vagina promoted the ability of MSP in post coital seminal plasma to kill *Candidia albicans*. This antifungal activity appeared to involve lysis of the organism’s membranes and to be mediated by a C-terminal fragment of MSP, which included a glutamic acid residue involved in the ability of the protein to form coordinate bonds with Ca^2+^. It appeared that MSP coordination of Ca^2+^ at neutral pH inhibited the antifungal activity of the protein but at low pH, electrostatic interaction between the ion and the C-terminal glutamic acid of MSP (pKa 4.1) decreased, facilitating the ability of the protein to kill *C. albicans*. Porcine MSP appeared to use a similar pH dependent antifungal mechanism, suggesting that it may be a widespread innate immune factor active against *C. albicans* and possibly helping to explain the low sexual transmission rate of vulvovaginal candidiasis in humans [37]. *C. albicans* was also reported to be susceptible to the pH dependent activity of LL-37 and its derivatives, KS-30, and RK-31, along with CRAMP [38], which is a murine homologue of LL-37 [136]. It appeared that KS-30, and RK-31 were produced by the proteolytic cleavage of LL-37 in the low pH environment of human sweat and that these pH conditions enhanced the ability all the peptides tested to kill *C. albicans* via permeabilisation of the fungal membrane. The use of animal models showed that although LL-37 and its derivatives were induced in murine skin in response to *C. albicans* infection, this induction did not confer subcutaneous resistance to the organism [38]. Based on these results, it was suggested that these peptides may be of primary importance in forming a barrier against fungal infections on the skin surface [38], given that the dysregulated production of LL-37 and its derivatives has been strongly associated with skin disease due to fungi and other microbes [137,138,139]. Hepcidin, (hep-25) is a human β-sheet hormone that has a well-established role in iron homeostasis [140] and has been shown to exhibit pH dependent antimicrobial activity [39]. In particular, acid conditions have been shown to enhance the activity of hep-25 and several of its isoforms, such as hep-20, against the fungal pathogen, *Candida glabrata* [40], along with a range of Gram-negative bacteria, such as Pseudomonas aeruginosa, and Gram-positive bacteria, including Enterococcus faecium [41,42]. Studies on *E. coli* suggested that the antibacterial action of these peptides involved membranolytic mechanisms which were enhanced under acid conditions due to the presence of histidine residues within their primary structure [43]. Based on these observations, it was suggested that with acidic pH, the increase in positive charge of histidine residues in hep-25 and hep-20 would promote their ability to target and lyse bacterial membranes, resulting in the death of the host organism [39]. Hep-20 has also been shown to be effective against drug resistant *C. glabrata* under the low pH and physiological conditions associated with the vagina, which led to the suggestion that the peptide may form the basis of novel therapeutics for the control of vaginal infections due to the organism [44]. Another group of human AMPs rich in histidine residues are the histatins (hst), which are salivary peptides with antiviral, antibacterial and antifungal activity [141,142,143,144,145,146] and protecting the oral cavity from fungal pathogens appears to be to the primary role of these peptides [147,148]. The pH of the oral cavity is mainly governed by that of saliva, which has a range that generally varies between pH 5 and pH 8 [149,150] although significantly lower pH values can occur on the surface of teeth due to the metabolic activities of cariogenic microorganisms [151], which is able to promote the growth of fungi and form mixed species biofilms [152,153]. Low pH appears able to enhance the antifungal activity of hst-1, hst-3 [154] and hst-5 [45], which, for example, enhances the positive charge carried by hst-5 and facilitates its translocation into fungal cells to attack intracellular targets [45]. However, a detailed description of the antifungal activity of these peptides is lacking and a number of mechanisms have been proposed [141,143,144,145,146], such as complex formation with iron to interfere with the cellular metabolism of the metal in fungi, such as *C. albicans* [155]. Studies on human lactoferrin, which is a multifunctional iron-binding protein [156] found that low pH enhanced the ability of sub-lethal levels of the protein to kill *C. albicans*, through multiple mechanisms, including dissipation of the proton motive force (PMF) across the CM of the organism [47]. This pH effect was attributed to increased electrostatic interactions between the peptide and anionic components of the *C. albicans* membrane, thereby enhancing the ability of lactoferrin to partition into fungal CM and generate lesions associated with PMF dissipation and the peptide’s antifungal action [47]. Sub lethal levels of lactoferrin have also been shown to be effective against biofilms of P. aeruginosa [157] and the protein was found to be able to synergize the activity of other antimicrobials against biofilms formed from P. aeruginosa and methicillin resistant *Staphylococcus aueus* (MRSA) [158,159]. It was proposed that this anti-biofilm activity was due to the iron-binding properties of the protein [157] but this does not appear to be the only mechanism involved in this activity [160] and acidic pH is strongly associated with these sessile microbial communities [161]. Lactoferricin B, which is a potent AMP derived from bovine lactoferrin [162], has also been shown to possess pH dependent antimicrobial activity, killing bacteria and *C. albicans* at low pH via mechanisms that appeared to involve membranolysis [56,57]. Low pH was also found to enhance the activity of platelet microbiocidal proteins, which were isolated from leporine platelets and showed activity against *Staphylococcus aureus*, *E. coli*, and *C. albicans* [53]. These results strongly supported the mounting evidence that platelets serve multiple important roles in host defense against infection, including the localized release of AMPs and other antimicrobial factors in response to microbial colonization and other stimuli [163]. The pH dependent antimicrobial mechanisms of platelet microbiocidal proteins were not further characterized but other studies showed that the antifungal and antibacterial activity of a number of these AMPs involved dissipation of the PMF across the cytoplasmic membrane of target organisms, which was able to synergize the activity of conventional antibiotics [164]. These results parallel those described above for human lactoferrin, allowing the speculation that low pH enhances the ability of some leporine platelet microbiocidal proteins to interact with membranes of *C. albicans* and generate lesions associated with PMF dissipation and the peptide’s antifungal action. Other leporine AMPs shown to possess pH dependent activity are the defensins, NP1 and NP2, which were found to permeabilize the outer membrane of *P. aeruginosa* most efficiently at low pH although these peptides were ineffective against the organism under these pH conditions. These AMPs are present in leporine macrophages and it was suggested that this pH dependent membranolytic activity may synergize the antibacterial action of other defense molecules under the acid conditions associated with phagocytosis [54,55].

**Figure 3:**
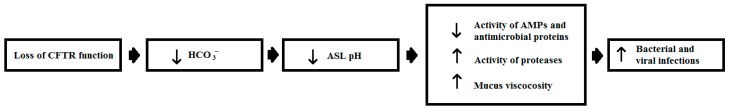
The pH of airway surface liquid and the pathogenesis of cystic fibrosis. Figure 3 was revised from [165] and shows a scheme for how changes in airway surface liquid (ASL) pH may influence the pathogenesis of cystic fibrosis (CF). In CF, the loss of cystic fibrosis transmembrane conductance regulator (CFTR) function results in decreased HCO_3_^−^ conductance across airway epithelial cells and leads to low pH in the ASL. Under these pH conditions, ASL AMPs, such as LL-37, HNP-1, HBD-1 and lactoferrin, and antimicrobial proteins, such as lysozyme, surfactant protein A and surfactant protein D, have reduced activity. Lower pH also leads to the increased viscosity of mucins, decreased ciliary beat frequency, impaired phagocyte function and depleted ASL volume. These effects lead to a decrease in the antimicrobial efficacy of the ASL and subsequently contribute to increased respiratory infections in the CF airway, caused by both viral and bacterial pathogens [165,166,179,185].

A particularly important case of human AMPs with activity that can be influenced by pH is that found in the airway surface liquid (ASL) of individuals with cystic fibrosis (CF) [165,166], which is a lethal genetic disorder characterized by viscous mucus and bacterial colonization of the airways [167]. In the mammalian respiratory system, the ASL represents a first line of pulmonary defense by forming the interface between the environment and the host organism and helping to protect against the action of inhaled and aspirated bacteria by producing a variety of antimicrobial molecules [168,169]. These ASL molecules include AMPs, such as LL-37, HNP-1, HBD-1 and lactoferrin, along with antimicrobial proteins, such as lysozyme, surfactant protein A and surfactant protein D [165,166]. Many of these antimicrobial molecules also contribute to the pulmonary innate immune system by adorning lattices of extracellular DNA, chromatin, enzymes and other proteins to form neutrophil extracellular traps (NETs). These DNA complexes are released in response to the presence of microbial pathogens and provide a mechanism for the localized concentration of effector molecules. NETs have been reported to be able to eradicate microbial pathogens using a variety of mechanisms, including the action of antimicrobial attachments and proteolytic degradation, as well as neutralizing their activity by forming a physical barrier that prevents the dissemination of these pathogens [166,170,171,172]. More recent studies have shown that the Human Short Palate Lung Nasal Epithelial Clone 1 (SPLUNC1), which is a protein expressed in the upper airways of the lung, plays multiple roles in pulmonary innate immunity. These roles include: the direct inhibition of bacterial growth, the prevention of microbial biofilm formation and the regulation of other AMPs and antimicrobial proteins, such as LL-37, HBD-2 and lysozyme [173,174,175,176]. However, in CF, mutations in the cystic fibrosis transmembrane conductance regulator (CFTR) gene leads to reduced HCO_3_^−^ secretion and produces an abnormally acidic pH in ASL [177,178], which studies on humans and animal models have suggested can negatively affect the efficacy of ASL antimicrobial molecules and predispose CF airways to microbial infection (Figure 3) [165,179]. For example, recent studies on a porcine CF model showed that the low pH of the ASL inhibited the activity of LL-37, lactoferrin and other AMPs when directed against *S. aureus* and *P. aeruginosa* [180,181], which are known, major CF pathogens [182,183]. These acid conditions also reduced the ability of ASL AMPs to synergize their activities when in combination with each other and with antimicrobial proteins, such as lysozyme [180,181]. It has been further proposed that conditions of low pH in CF airways could reduce the efficacy of AMPs and antimicrobial proteins that adorn NETs [170] along with the antimicrobial and other biological activities of SPLUNC1 [179,184]. The mechanisms by which the low pH impairs the activity of AMPs and antimicrobial proteins in CF airways are currently unclear but it has been suggested that these mechanisms include a variety of contributions. For example, it has been proposed that low pH in CF airways may mediate the degradation of AMPs via the activation of host proteases, such as cysteine cathepsins, and microbial enzymes, such as aureolysin of *S. aureus* and elastase of *P. aeruginosa*, and the immobilization of AMPs through binding to mucins, which are large, anionic glycoproteins and the primary component of mucus. It has been further proposed that low pH in CF airways may induce conformational changes in AMPs that reduce the ability of these peptides to bind microbial membranes and cell wall components, such as lipid II [165,166,170,180,181,185]. In addition to reducing the activity of AMPs, low pH appears able to negatively impact on other defense factors of the ASL; for example, by increasing the rheological properties of secreted mucins, decreasing ciliary beat frequency, impairing phagocyte function and depleting ASL volume [165,166,179,185]. Based on these observations, it has been proposed that connections between the loss of CFTR, reduced ASL pH, and impaired CF host defense function could provide a paradigm for the identification of new therapeutic targets and strategies to reduce the morbidity associated with CF lung disease [165].

A number of anionic AMPs with pH dependent activity have been identified in humans, including DCD-1(L), which is proteolytically cleaved from dermcidin, which is also an anionic peptide and found in human sweat [9,48,186,187]. DCD-1(L) is characterized by its broad range antimicrobial activity, killing fungi, such as *C. albicans*, as well as Gram-positive bacteria, including MRSA, Gram-negative bacteria, such as *Salmonella typhimurium*, and acid fast bacteria including rifampin- and isoniazid-resistant *Mycobacterium tuberculosis* [188,189,190,191,192,193]. The peptide appears to exhibit pH dependent antibacterial action [188] whereby low pH induces the peptide to adopt α-helical structure on the bacterial membrane surface, leading to ion channel formation via Zn^2+^ stabilized DCD-1(L) oligomers and death of the host organism through membrane disruption [48,49]. A more recent study gives general support to this model and suggested that under acid conditions, the negative charge on DCD-1(L) becomes neutral, which facilitates membrane partitioning and Zn^2+^ dependent membrane channel formation via either a barrel-stave pore or a toroidal pore type mechanism [50]. Another example of AAMPs with pH-dependent activity are kappacin A and kappacin B, which are classed as food peptides and appear to be cleaved from κ-casein in bovine milk by digestion with chymosinin in the human stomach [194]. These peptides exhibit potent activity against a range of Gram-positive and Gram-negative bacteria, including *Streptococcus mutans*, *Porphyromonas gingivalis* and *Actinomyces lundii* [51,52], which make a major contribution to supragingival dental plaques [195,196]. Characterization studies showed that the antibacterial mechanisms used by kappacins involved the pH-dependent lysis of microbial membranes, which was enhanced by acid conditions [52]. The active region of kappacins included a phosphorylated serine residue that was essential for antibacterial activity [52,197] and interestingly, these peptides showed significantly different pH optima for this activity that resulted from a single residue difference in the sequence of their active regions [51,197]. Kappacin A showed the highest antibacterial activity of the two peptides and possessed an aspartic acid residue in its active region, which was replaced by an alanine residue in the corresponding location of kappacin B [51,197,198]. The functional significance of this difference in sequences is not known although it has been proposed that the additional negative charge possessed by kappacin A may enhance its ability to bind metal ions [9]. It had been demonstrated that the presence of Ca^2+^ and Zn^2+^ ions enhanced the antibacterial activity of kappacins and it was suggested that these ions may form a cationic salt bridge between kappacins and anionic components of the bacterial membrane, thereby facilitating membrane binding and antibacterial action [197]. It has been proposed that the membrane interactive conformation of these peptides may be a proline-kinked amphiphilic α-helix but conformational changes observed in the peptide in the presence of membrane mimic could not be clearly assigned to any particular secondary structures and the structure of kappacins remains unclear [9,52,197,198].

### 2.4. Marine Invertebrates

Myticin C (myt C) exists as a number of isoforms in the bivalve mollusc, *Mytilus galloprovincialis* [199,200,201] and has been shown to possess activity against fish viruses, including Hemorrhagic Septicaemia virus and Infectious Pancreatic Necrosis virus [202]. Several studies were conducted on the antimicrobial activity of a reduced form of myt C, (myt Cc, [58,59]) based on the proposal that the endogenous reduction of cysteine-stabilized AMPs to produce peptides with higher levels of antimicrobial activity may form part of some innate immune systems [116,117]. It was found that the antiviral activity of myt C, myt Cc and derivatives of both these peptides was enhanced by low pH. These studies also showed that only under acid conditions did these various peptides possess activity against Gram-positive bacteria and Gram-negative bacteria. Structure function studies on *E. coli* suggested that the antimicrobial action of myt C and its variants was membranolytic and involved low pH mediated increases in their levels of α-helicity and β-hairpin elements within their molecular architecture [58,59]. In addition to their pH dependent antimicrobial activity, both myt C and myt Cc possessed chemotactic activity and appeared to be the first chemokine/cytokine-like molecules identified in bivalves [59,202]. These results added to the increasing evidence that AMPs can serve as cytokines [90,203] and interestingly, it is also becoming clear that these latter peptides are able to exhibit antimicrobial activity [204,205] that is enhanced by low pH [206,207]. Molluscs are also a source of histidine containing AMPs with pH dependent activity, such as the peptide, KPS-1, which was isolated from, *Atrina pectinate*, and under acid conditions, inhibited the growth of a range of Gram-negative bacteria, including *P. Aeruginosa*, *S. typhimurium* and *Enterobacter sakazakii* [60]. Ci-PAP-A22 and Ci-MAM-A24 are representative peptides of the Ci-PAP and Ci-MAM families of AMPs from the solitary tunicate (Sea squirt), *Ciona intestinalis* [61,62], which appear to be produced in haemocytes and granulocytes of the organism. [208,209]. Both peptides were found to be predominantly α-helical and to possess antimicrobial activity [64,209,210] that appeared to have a pH dependency with optima that varied according to the target microbes [61,62,63,64]. In the case of Ci-PAP-A22, the activity of the peptide against fungi, Gram-negative and Gram-positive bacteria was enhanced by neutral pH except for *B. megaterium* which was more efficiently killed by Ci-PAP-A22 at acid pH [61]. In contrast, Ci-MAM-A24 showed enhanced activity against *B. megaterium*, *B. subtilis*, *E. coli* and *P. aeruginosa* at low pH but neutral pH optima for action towards *S. aureus*, *Staphylococcus epidermis*, *Serratia marsecens* and *Klebsiella pneumoniae*. The peptide was also found to exhibit pH independent antimicrobial activity, killing comparable levels of *Yersinia enterocolitica* and fungi under low and neutral conditions of pH [62]. The antimicrobial activity of both Ci-PAP-A22 and Ci-MAM-A24 appeared to involve a membranolytic mechanism that had characteristics consistent with a ‘carpet’ or ‘toroidal pore, type model (Table 1). To help explain the differences in pH dependent antimicrobial activity shown by these peptides it was suggested that histidine mediated variation in their positive charge may facilitate optimal membrane interaction on a species-specific basis [61,62] and of course varying lipid composition will mean differing bacterial systems exhibit different changes to key parameters such as lipid packing at varying pH. Interestingly, Ci-MAM-A24 was found to be more potent than Ci-PAP-A22 and appears to be the first AMP reported to kill an intra-amoebic pathogen [63]. It was demonstrated that Ci-MAM-A24 was able to kill *Legionella pneumophilia*, which is a Gram-negative parasite responsible for Legionnaire’s disease [211], whilst the organism was replicating intracellularly in *Acanthamoeba castellani* [63]. It is well established that *A. castellani* acts as a vector for this bacterium [212,213], which efficiently replicates in the acidic environment of host amoebal phagosomes [214,215]. Ci-MAM-A24 was also able to kill Mycobacteria, in murine macrophages [210] and these acid fast bacteria are known to replicate in the acidic compartments of these host cells [216]. Given the pH dependent antimicrobial activity of the peptide, it is tempting to speculate that the ability of Ci-MAM-A24 to kill these various bacterial parasites was potentiated by the low pH of their host cell environments. Clavaspirin, clavanins and styelins were isolated from another solitary tunicate, *Styela clava* and these pH dependent AMPs were found to be rich in both histidine and phenylalanine residues [68,69]. In general, it was found that clavaspirin and clavanins possessed pH dependent antibacterial and antifungal activity [65,66,67,76] with low pH, enhancing the ability of these AMPs to adopt α-helical structure and permeabilize the membranes of these organisms [68,69]. It appeared that the protonation of histidine residues under low pH conditions promoted the ability of these AMPs to target microbial membranes whilst the presence of their glycine and phenylalanine residues provided them with the conformational flexibility and structural hydrophobicity to facilitate bilayer partitioning [70,71,72,73,74,75]. Styelins, which are rich in phenylalanine residues, were found to show activity against both human bacterial pathogens and marine bacteria, such as *Psychrobacter immobilis* and *Planococcus citreus*, [68,217]. The best characterized of these AMPs is styelin D, which possesses α-helical structure and is highly unusual in that it contains twelve post-translationally modified residues [77]. For example, the peptide contained multiple bromotryptophan residues, which are found in the AMPs of other marine organisms [218,219,220,221,222] and play an important role in the life of sea sponges and lower marine invertebrates [223]. Styelin D’s post-translationally modified residues enhanced the peptide’s membranolytic action at low pH but only against Gram-positive bacteria. It was suggested that a role for these extensive modifications may be in preserving activity against certain organisms under the acid conditions found in haemocytes of *S. clava* where the styelins are active [77].

### 2.5. Terrestrial Invertebrates

Hebraein is produced by the tick, *Amblyomma hebraeum* [224], and showed acid pH optima for its activity against *E. coli*, *S*. *aureus* and the fungus, *C. glabrata* [78], a major cause of vulvovaginal candidiasis in diabetics [225]. The peptide possessed an α-helical structure except for a short C-terminal extension containing multiple histidine residues, which appeared to be required for activity against these organisms [78]. Based on these observations, it was suggested that the acidic pH induced in the physiological environment when a tick blood-feeds would increase the cationicity of hebraein and thereby its membrane interactivity and antimicrobial potency [78]. However, the activity of hebraein against *S. aureus* appeared to be independent of this histidine cluster and *C. albicans* was not susceptible to the action of the peptide, suggesting that it possessed a variety of antimicrobial mechanisms, which were influenced by the target organism [78]. Interestingly, these latter studies showed hebraein to possess homology and structural similarities to microplusin, which is a Cu^2+^ chelating peptide isolated from another arachnid, the cattle tick, *Riphicephalus microplus*, with a broad range of antimicrobial activity [226,227,228]. Studies on the Gram-positive bacterium, *Micrococcus luteus*, and the fungus, *Cryptococcus neoformans*, suggested that Cu^2+^ chelation involving histidine residues promotes the antimicrobial activity of microplusin by depriving vital cellular processes of the ion, such as haeme-copper terminal oxidases that contribute to cell respiration [228,229,230]. Amoebapores are a family of cystein stabilized antimicrobial proteins with α-helical structures that are found in the cytoplasmic granules of the protozoan parasite of primates, *Entamoeba histolytica* [79,81,231], and interestingly there is evidence to suggest that amoeba-like peptides may have been amongst the first eukaryotic AMPs to emerge [232]. Amoebapore A is the best characterized of this family of proteins and has been shown to exhibit pH dependent activity against Gram-positive organisms, such as *M. luteus*, and Gram-negative bacteria, including *E. coli* [79,80,83], which appears to involve pore formation in the membranes of target organisms [80]. Both pore formation and the antibacterial activity of the protein were enhanced by low pH, which appears to derive from an increased ability of amoebapore A to self-associate and form oligomers with some similarities to a barrel-stave pore type mode of action [79,80,81]. Elucidation of the structure of amoebapore A indicated that a C-terminal histidine residue acted as a molecular switch that triggers the formation of active dimers from inactive monomers, which leads to the construction of oligomeric pores in target cell membranes, [82]. In addition to amoebapore A, two isoforms of the protein, amoebapores, B and C, are included in the amoebapore family and all three isoforms differ markedly in their primary structure and spectra of antibacterial activity, synergising their combined efficacy. Amoebapores, B and C are believed to have similar antibacterial mechanisms to amoebapore A and the acidic pH optima of these proteins is consistent with the low pH conditions encountered in the amoebic intracellular vesicles, which form their site of action [79,81,231]. More recently, acanthaporin, which is another protozoan protein with pH dependent antimicrobial activity was described in the *Acanthamoeba culbertsoni*. At neutral pH, acanthaporin appears to exist as an inactive dimer but low pH triggers the histidine mediated production of monomers and the formation of membrane pores, which promoted the activity of the peptide against a variety of bacteria [84]. Caenopores, also known as saposin-like proteins (SPP), are cystein stabilized helical proteins that are found in the nematode, *Caenorhabditis elegans* [89,232,233,234,235] and are distantly related to amoebapores with which they share structural and functional features [85,88,236,237]. Many of the genes encoding SPP proteins in *C. elegans* are induced in response to microbial challenge [232] and several of their gene products have been reported to exhibit pH dependent antimicrobial activity, including SSP-1 [85,86], SPP-3 [87], SPP-5 [88] and SPP-12 [86]. These studies showed that that low pH enhanced the ability of caenopores to kill a wide range of microbes, including Gram-negative bacteria, such as *E. coli*; Gram-positive bacteria, including *Bacillus thuringiensis;* yeasts, such as *Saccharomyces* *cerevisiae*; and amoebae, including *Dictyostelium discoideum*. For each of these proteins, antimicrobial activity appeared to be based on an ability to form pores in membranes of target organisms under acid pH conditions and it was suggested that this ability was mediated by the multiple internal histidine residues possessed by caenopores. Due to these residues, the positive charge of these proteins is enhanced under acid conditions, increasing the potential for interaction with anionic components of microbial membranes and possibly mediating pore formation, as described for amoebapores [86,87,88,89,236,237]. It was observed that the pH dependent activity of these proteins would appear to reflect the pH conditions at the site of their functional action, such as SPP-1 and SPP-5, which are active in the acidic environment of the *C. elegans* intestine [88,89,236,237].

## 3. Potential Applications of pH Dependent Antimicrobial Peptides and Proteins 

In response to the growing demand for new antibiotics with novel mechanisms of action, the number of AMPs and antimicrobial proteins entering clinical trials is accelerating [12] and included within these antimicrobial molecules are a number that have been reviewed here (Table 2). Currently, the only pH dependent anionic AMPs that appear to have been commercially developed are kappacins. Based on their activity against oral pathogens [51,52], preparations including these peptides and zinc have been patented [238] and are available as a dental care products [194,198]. It has also been shown that these peptides exhibit increased antimicrobial activity in foods with high calcium contents [194], which, taken with the history of the safe use of κ-casein, led to the proposal that kappacins may be used as a preservative [239]. In the case PD-3-7, epimers of this peptide appear to be the only amyloid forming amphibian anionic AMPs so far reported and have the potential to progress understanding of the role of residue chirality in the formation of disease-related amyloid and aid the design of amyloid-based nanomaterials [125]. The development of functional amyloids as novel nanostructure materials for multiple purposes, such as drug delivery and tissue repair/engineering, is a growing area of technology [240,241], and recently techniques have been developed to detect epimeric AMPs in the complex skin secretions of frogs and toads [242].

The most researched of the cationic AMPs reviewed here for potential medical development is LL-37, which is a prospective broad range antimicrobial agent that is also able to induce wound healing and angiogenesis as well as modulate apoptosis [243]. This potential is, however, limited in some cases by the pleiotropic effects of the peptide [244]. For example, the peptide shows a variation in its sensitivity to cancer types, promoting proliferation, migration, and tumorigenesis in breast, lung, and prostate cancers through receptor signaling but suppresses proliferation and induces apoptotic and autophagic cell death in gastric cancer, colon cancer, and T-cell leukemia [107]. However, wound treatment is a globally prevalent and economic burden, which makes the pleiotropic ability of LL-37 to exert healing properties and combat multiple microbial pathogens an attractive platform that has been used to develop potential therapeutic strategies for wound treatment [245]. For example, a clinical phase I/II study conducted by Pergamum on LL-37 led to a patent [246] and showed that topical application of the peptide was safe and enhanced wound healing in patients with chronic venous leg ulcers and diabetic patients suffering from infected wounds [245,247]. Wound infection is a major complication in diabetic patients and in particular, infected foot ulcers is one of the most serious and frequent of these complications, which accounts for over 50% of all lower limb amputations performed on these patients [248]. More recently, several studies have developed biodegradable drug delivery system that facilitated the controlled sustained release of LL-37 and other wound healing agents, such as lactate and serpin A1, from nanoparticles. LL-37 and these agents acted synergistically in the treatment of full thickness excisional wounds, significantly promoting wound closure, reducing bacterial contamination and enhancing anti-inflammatory activity. These systems offered several advantages over therapies commonly used to treat chronic wound infections, which are often limited due to factors, such as the lack of controlled delivery and the depth of skin infections [249,250]. A number of LL-37 related peptides have also shown the potential for therapeutic development [243,251], such as OP-145, which was developed by OctoPlus, and when the peptide was included in cream formulations for nasal application, these preparations were found to be efficacious in the eradication of MRSA carriage [252]. The anterior nares are the main reservoir for colonization by *S. aureus* and the nasal carriage of MRSA is an important risk factor for subsequent infection and transmission of this pathogen, which has led to intensive efforts to identify agents able to efficiently reduce MRSA colonization [253]. The completion of phase I/II clinical trials by OP-145 also showed that the peptide was safe and efficacious as a treatment for chronic otitis media, or chronic bacterial middle-ear infection (Table 2) [254]. This disease afflicts millions of people worldwide and is highly recalcitrant to treatment by conventional antibiotics, which is now known to be primarily due to bacterial biofilms [255]. Another derivative of LL-37, 60.4Ac, has also proven to be beneficial in the treatment of patients with otitis media [245] and more recently the peptide showed the potential for development as a novel local therapy to treat patients with burn wounds infected with multidrug-resistant bacteria, including MRSA [256]. Burn wounds are one of the most common and devastating forms of trauma and the infection of these wounds by drug resistant bacterial pathogens is rapidly becoming a serious therapeutic challenge in the care of burn patients [257].

Histatins and their derivatives show the potential for a wide range of therapeutic and biotechnical application [141], particularly in the field of dentistry and bio-dental research [258]. For example, the hst-5 derivative, JH8194, is a promising candidate to act as a surface substrate in dental implants to prevent peri-implantitis and peri-implant mucositis whilst decreasing infections [259,260]. A major focus in the medical development of histatins has been in the preparation of formulations to treat oral diseases and infections [141]. For example, highly effective hydrogel delivery systems for the topical and oral application of hst-5 have been developed for the treatment of oral candidiasis [261], which is the most common opportunistic fungal infection in immunocompromised populations [262]. High potential for the topical treatment of this fungal condition was also demonstrated when derivatives of hst-5 were conjugated to spermidine and tested on immunocompromised murine models [263]. Compared to hst-5, these conjugates exhibited a higher clinical half-life, enhanced uptake into *Candida* cells, and greater candidacidal efficacies, and were proposed to be viable alternatives to azole antifungals [263], which are commonly used to treat oral candidiasis [262]. A compound derived from hst-5 and hs1-3, P-113 (PAC-113), developed by Pacgen, was evaluated in Phase 1/II clinical studies for the treatment of both oral candidiasis and gingivitis, and was found to be safe and effective in the treatment of both conditions (Table 2) [12]. Gingivitis is the most common form of periodontal disease, affecting up to 15% of the adult populations worldwide and primarily due to *Porphyromonas gingivalis*. Untreated, the condition can lead to periodontitis, the chronic destruction of connective tissues, and ultimately result in loss of teeth [264]. P-113 has been patented [265] and most recently, it has been shown that the candicidal efficacy of the peptide was greatly enhanced when it was modified by coupling to other AMPs and their derivatives [144]. Another histatin, hist-1, was conjugated to a silver metallopharmaceutical and the conjugate was found to have wound healing properties coupled to potent activity against bacteria, which included MRSA, indicating the potential for development of novel multifunctional therapeutics [266]. Clavanins are attractive candidates for development as drugs against bacteria associated with sepsis, which is rapidly becoming a problematic nosocomial infection [267], and recently developed nanoparticle formulations of these peptides showed high promise as a drug against polymicrobial sepsis with morphological characteristics suitable for administration via injection [76]. Derivatives of clavanins have also been developed to combat biofilms formed by *S. mutans*, which is a major contributor to dental plaque and one of the major etiological factors involved in causing caries [268]. Dental caries are one of the most prevalent, preventable infectious diseases affecting humans and are recognized as the primary cause of oral pain and tooth loss [269].

The major medical development of the antimicrobial proteins reviewed here appears to be lactoferrin, which, as described above, is an iron binding protein but, like LL-37, is pleiotropic and also displays a broad range of antimicrobial activity using a number of mechanisms, which includes the release of derivative AMPs via hydrolysis by proteases [162,270,271]. Lactoferrin and its related peptides shows the potential for a number of clinical uses, ranging from wound healing and the detection of bacteria to the treatment of microbial infections both alone and in combination with other clinically relevant agents [272]. A full description of these medical uses is beyond the scope of this review but lactoferrin and its derivatives have featured in multiple clinical trials [160,272,273] and have numerous entries in a recently constructed database of bioactive peptides derived from milk proteins [274]. As major examples, lactoferrin and its derivatives have been extensively investigated as potential drugs for the treatment of common viral infections including the common cold, influenza, viral gastroenteritis and herpes [275] whilst the inhibitory effects of these proteins and peptides against the proliferation of multiple cancers, has suggested a potential role in cancer prevention [276]. It is well established that many AMPs and antimicrobial proteins have anticancer activity that generally appears to involve mechanisms of membranolysis that are similar to those used by these molecules in their action against microbes [277,278], which in some cases shows pH dependence [98], as recently described [279,280]. Advanced clinical trials have shown that the administration of lactoferrin has no significant side effects and that the protein has efficacy in treating iron deficiency anemia in pregnant women [281], sepsis in premature neonates, which is a common and severe complication in new-born infants [282] and infections due to *Helicobacter pylori*, which is causally associated with gastritis and peptic ulcer diseases [283]. A major example of the medical potential of lactoferrin is the development of ALX-109 by Alaxia, which is a combination of the protein and hypothiocyanite for the treatment of CF [160]. This drug combination has been granted orphan drug status by American and European licensing agencies and has been shown to enhance the ability of conventional antibiotics to eliminate biofilms of *P. aeruginosa* growing on CF airway epithelial cells [284]. Derivatives of lactoferrin, have also shown the potential for therapeutic development such as hLF(1-11), which was developed by AM Pharma, and in clinical trials the peptide was safely injected into neutropenic stem cell transplantation patients [160]. Neutropenia is defined as a reduction in the absolute number of neutrophils in the blood circulation, predisposing individuals to severe or fatal infections [285], and currently, hLF(1-11) awaits development for the prevention of bacteremia and fungal infections in immunocompromised individuals (Table 2) [273]. Lactoferricin B is cleaved from the N-terminal region of bovine lactoferrin under acid pH conditions and has an extremely wide spectrum of antimicrobial activity against bacterial, fungal and parasite species as well as showing anti-catabolic and anti-inflammatory effects [162,270,271]. Based on these abilities, this peptide has featured in numerous preclinical trials and shows the potential for a variety of therapeutic purposes, including the treatment of ocular infections, osteo-articular gastro-Intestinal and dermatological diseases, along with applications in veterinary practice and the food industry [272]. The commercial importance of lactoferrin and its derivatives is perhaps underlined by the fact that the recombinant human protein has been expressed in transgenic cattle to provide the large-scale production of lactoferrin for pharmaceutical use [286]. The recombinant protein has also been expressed in microbes and higher plants in the search for bioreactors with the capacity for large-scale production, which, led to lactoferrin expression also being used as a tool for the enhancement of plant resistance to pathogens [286].

## 4. Discussion

AMPs and antimicrobial proteins with pH dependent action against microbes appear to receive relatively little attention in the literature but, as this review has shown, these molecules are produced by a diverse spectrum of eukaryotes, including: vertebrates, such as fish, humans, horses, cattle, rabbits, guinea pigs, mice, frogs and toads, as well as invertebrates, such as ticks, parasites, worms and mollusks (Table 3). Around two thirds of the molecules reviewed here are cationic AMPs and antimicrobial proteins with most of those that remain possessing net negative charges [287]. It is generally recognized that the incidence of anionic antimicrobial molecules is low and that, in general, their occurrence appears to be a strategy to synergize the antimicrobial activity of their cationic counterparts [9,288]. For example, the proteolytic processing of the sweat borne peptide, dermcidin, to yield DCD-1(L), described above, also produces a number of other anionic AMPs, such as SSL-46 (net charge –2) and LEK-45 (net charge -2) [289]. These sweat-derived anionic AMPs are continually secreted and are believed to synergize the activity of cationic AMPs in the constitutive innate defense of human skin by modulating surface colonization by microbes rather than responding to injury and inflammation as observed for inducible peptides, such as LL-37 [186].

The pH dependence of the antimicrobial molecules reviewed here was found to vary with pH with some, such as E2EM-lin, exhibiting high pH optima (Table 4) whilst others, such as Ci-PAP-A22 and Ci-MAM-A24, exhibited optima at either neutral or acid pH depending on the target organisms [61,62,63,64]. Again depending on the target microbes, several antimicrobial molecules, including the latter peptides and psiorasin, showed the ability to employ both pH dependent and pH independent activity [34,35,36,61,62,63,64]. However, most of the AMPs and antimicrobial proteins reviewed here exhibited low pH optima, which is consistent with the acidic pH found at their sites of action, particularly the skin [131,290]. Consistent with these observations, the major structure/function relationships that promote the pH dependent activity of the antimicrobial molecules reviewed here are those involving amino acid residues that become protonated under acid conditions, including histidine, aspartic acid and glutamic acid residues. Under these pH conditions, the protonation of these residues will have the overall effect of increasing the cationicity or decreasing the anionicity of the parent molecule, thereby enhancing its ability to target and interact with negatively charged components of microbial membranes. Typical examples include hebraein [224] and clavanins [70,71,72,73,74,75], and in the case of Ci-PAP-A22 and Ci-MAM-A24, it appears that the histidine mediated variation in the cationicity of these peptides facilitates optimal interaction with target microbial membranes on a species-specific basis [61,62]. However, given the high incidence of histidine residues in the antimicrobial molecules reviewed here, it is worth noting that the possession of these residues is not necessarily sufficient for a pH dependent mode of antimicrobial action. This point is well illustrated by Pc-pis, from the yellow croaker, *Pseudosciaena crocea*, which includes a number of histidine residues in its primary structure and displays pH independent antimicrobial activity. However, the addition of a histidine residue to its sequence generated a peptide with antimicrobial activity optimal at low pH and a wider spectrum of antimicrobial activity [291].

A second major structure/function relationship for histidine, aspartic acid and glutamic acid residues in the antimicrobial action of pH dependent AMPs and proteins reviewed here is to facilitate the binding of metal ions. For example, the binding of Ca^2+^ by MSP at low pH potentiates the activity of the peptide by alleviating inhibitory mechanisms that are mediated by the ion [37] and metal ion binding by histidine residues appears able to promote microbial death through depletion of these ions for a number of antimicrobial molecules, such as histatins [141,143,155]. In contrast, the binding of metal ions appears to potentiate the activity of some antimicrobial molecules reviewed here by promoting their capacity to form peptide–membrane or peptide–peptide salt bridges and thereby disrupt microbial membranes, as proposed for kappacins [197] and DCD-1(L) respectively [50]. However, the most common structure/function relationships for histidine, aspartic acid and glutamic acid residues in the antimicrobial action of the molecules reviewed here are to directly promote the disruption of target microbial membranes. For example, in the case of several antimicrobial proteins, the protonation of histidine residues appears to be a molecular switch that initiates oligomerisation and the formation of discrete channels or pores by the protein, as in the case of acanthaporin [84]. In some cases though, histidine, aspartic acid and glutamic acid residues appear to play multiple roles in promoting the activity of their parent antimicrobial molecules. For example, the N-terminal regions of gad-1 and gad-2 include a number of sequential histidine pairs that appear to be important to their ability for lipid targeting and interaction, channel formation and thereby the disruption of microbial membranes at low pH [29,30,94,99,100].

A further major structure/function relationship involved in the mechanisms of the antimicrobial molecules reviewed here is pH related conformational change in α-helical architecture, which is by far the most common secondary structural element identified in these AMPs and antimicrobial proteins. Indeed, it is well established that histidine, glutamic acid and aspartic acid residues have a strong potential for α-helical formation that is enhanced by low pH [98,292]. The pore forming antimicrobial proteins reviewed here are strongly α-helical (Figure 2) and it is known that changes to the levels of α-helical architecture possessed by these proteins are enhanced by low pH, which promotes their pore forming mechanisms and are key to their ability to kill microbes [35,82,84,89]. A full description of these conformational changes is beyond the scope of this review but as an example, the protonation of C-terminal histidine residues by low pH promotes conformational changes that lead to the construction of hexameric membrane pores via the formation of active dimers from inactive monomers in the case of amoebapores [79,81,231] caenopores [85,86,87,88,89] and psoriasin [34,35,36]. The pore forming mechanism of acanthaporin shows similarities to those of these latter proteins and also results in the formation of hexameric membrane pores. However, in the case of acanthaporin, the low pH mediated protonation of C-terminal histidine residues promotes conformational changes that induce pore formation via the formation of active dimers from inactive monomers [84]. Strictly, based on is size, lactoferrin is an antimicrobial protein, but it is often classified with AMPs due to its ubiquity in body fluids and its ability to kill bacteria using membrane interactive mechanisms with similarities to those of these latter peptides [90]. However, lactoferrin was first characterized as an iron binding protein and sequestration of the metal was initially believed to form the basis of its antibacterial mechanism although the protein is now known to use multiple iron-independent mechanisms in its activity against microbes [162,293].

In relation to the AMPs reviewed here, low pH generally increased their levels of α-helical secondary structure and thereby enhanced their capacity for membrane interaction and antimicrobial activity. However, alkaline conditions promoted maximal levels of α-helical structure in E2EM-lin, which appeared to promote monomer association, pore formation and membrane interaction at the peptide’s high pH optimum (Table 4, Figure 1). In contrast to these latter AMPs, gad-1 and gad-2 were found to possess minimal levels of α-helical structure under the low pH conditions that were optimal for their membrane interactions and antimicrobial activity [29]. These observations would seem to clearly indicate that pH dependent structural plasticity is an important factor in the antimicrobial mechanisms of many of the AMPs reviewed here. This form of structural plasticity would appear to be key to facilitating the appropriate balance between the amphiphilicity and hydrophobicity of these peptides that is required for their membranolytic action at optimal pH, as proposed for gad-1 and gad-2 [29]. Reinforcing this proposal, other amino acid residues have been reported to contribute to the structural plasticity of the α-helical AMPs reviewed here including glycine, phenylalanine and post-translationally modified residues. These residues appear to enhance the conformational flexibility and structural hydrophobicity of tunicate clavaspirin, clavanins and styelins for bilayer partitioning and antimicrobial action at their low pH optimum [68,69].

The antimicrobial mechanisms of several AMPs reviewed here appear to be described by models of membrane interaction, including variants of the carpet, toroidal pore and barrel-stave pore mechanisms. These models of membrane interaction differ fundamentally to the pore forming mechanisms of the antimicrobial proteins described above and were primarily proposed to describe the membrane spanning abilities of AMPs, which are generally up to 50 residues in length [23]. A number of novel antimicrobial mechanisms for AMPs have also been revealed by this review, such as that described for human lactoferrin, which at sub lethal levels appears to kill microbes via the pH dependent dissipation of microbial PMF [47]. The microbial PMF is an emerging potential target for the development of novel AMPs and antimicrobial proteins based on the fact that the temporary membrane perturbations caused by their action can have a large negative impact on bacterial metabolism, affecting a diverse array of cellular processes that depend upon the PMF [294,295,296,297,298,299]. This review has also described novel examples of pH dependent AMPs produced by the reduction of cysteine stabilized parent AMPs including myt Cc [59,202] and E2EM-lin (Table 4, Figure 1, [114,115]) and it has been recently shown that the free cysteines of reduced AMPs play an important role in their antimicrobial activity [116,117]. Moreover, it was speculated above that the antimicrobial activity of E2EM-lin may involve pore formation via self-association and interestingly, recent work has suggested that free cysteine residues may play a role in the antimicrobial activity of AMPs by facilitating the oligomerisation of these peptides [300]. Taken together, these reports show that AMPs with pH dependent antimicrobial activity contribute to the accumulating evidence that the endogenous reduction of cysteine-stabilized AMPs is a strategy used by hosts to generate novel peptides that enhance the efficacy of their antimicrobial capacity [116,117].

A number of the AMPs and antimicrobial proteins reviewed here, along with their derivatives, have been developed for multiple medical purposes, which, in some cases, has led to patents and the successful completion of clinical trials, and include kappacins, LL-37, histatins, lactoferrin and clavanins. Major examples of the application of these AMPs and proteins include the treatment of multiple cancers along with viral infections, such as the common cold; bacterial infections, including those associated with implants, otitis media, neutropenia and CF; and fungal infections, particularly, those detrimental to oral health. These AMPs and proteins also show the potential to induce wound healing, such as for diabetic patients and burn victims, and interestingly, a recent report has indicated that wound healing is accelerated by an acidic environment, which promotes a range of beneficial effects including increases in antimicrobial activity and the enhancement of epithelization and angiogenesis [301]. In general, the therapeutic administration of the AMPs and proteins involve topical application, such as the use of mouth washes, cream formulations and hydrogel delivery systems. These observations raise an interesting point in that most clinical trials to date involve the treatment of skin infections or the prevention of surface colonization by microbes, particularly sessile forms of these organisms, which, potentially, can indicate a wide variation in local pH conditions. A comprehensive understanding of the effect of pH on the antimicrobial activity of the molecule under development would therefore seem necessary. Nonetheless, this is not generally the case and data cited in the literature in relation to the antimicrobial activity of AMPs and proteins are usually determined under neutral pH conditions [133]. These observations clearly suggest that when characterizing the antimicrobial action of AMPs, the optimal pH for their action against individual microbes should be determined. This point is well illustrated by recent studies, which investigated the antimicrobial action of a range of synthetic AMPs and found that high pH inhibited the action of these peptides against fungi and Gram-negative bacteria but the opposite pH trend was observed for Gram-positive bacteria [302].

## 5. Conclusions

This review has shown that AMPs and proteins with pH dependent antimicrobial activity are increasingly being reported and that progress has been made in understanding the structure/function relationships and mechanisms underpinning this activity. This review has also shown that there has been considerable therapeutic development of pH dependent antimicrobial molecules to treat a variety of infections and other conditions. However, one of the biggest therapeutic and biotechnical developments of these antimicrobial molecules has been to provide guidance to the design of novel compounds with pH dependent activity against: bacteria [303,304], fungi [46,305,306] and cancer cells [307,308] as well as applications involving drug [309,310] and gene delivery [311,312]. As a specific example, most AMPs designed to target the low pH of tumor tissue are cationic and cytotoxicity to healthy tissue at physiological pH has often been an issue for these peptides [98,279,280]. To address this issue, a peptide based on magainin 2 from *X. laevis* was designed to possess a negative charge at neutral pH that switched to a strong positive charge at low pH for cancer targeting. Designated HE, this novel peptide, killed human renal adenocarcinoma at low pH via membranolytic mechanisms and was nontoxic towards healthy human cells across low and neutral pH conditions, making it a promising lead compound for cancer therapy [313]. As a further example, chronic infections due to *P. aeruginosa* are responsible for the majority of the morbidity and mortality in patients with CF and the persistence of these infections is largely due to the organism adopting a biofilm mode of growth, thereby acquiring high resistance to most antibiotics [314,315]. In response, the peptide, WLBU2, was designed and found to be able to prevent the biofilm formation by *P. aeruginosa* under the low pH and high salt conditions characteristic of the CF airway without negative effects on human airway epithelial cells. WLBU2 was also found to be able to synergize the action of commonly used antibiotics, such as tobramycin and meropenem, making the peptide an attractive proposition to help address the critical need for novel therapeutics that are able to suppress chronic CF lung infections [316]. Using another approach, it has also been proposed that increasing the airway pH in CF individuals by activating CFTR independent HCO_3_^−^ transport pathways or by inhibiting proton pumps could help prevent or reduce bacterial and viral infections associated with the disease [165,180,181]. Nonetheless, this review has shown that many pH dependent AMPs and antimicrobial proteins have yet to be fully characterized and it is proposed that these antimicrobial molecules merit far more research attention than they currently receive. Indeed, pH dependent AMPs and antimicrobial proteins appear to represent an untapped source of novel biologically active agents that is awaiting full exploitation and could aid fulfillment of the urgent need for alternatives to conventional antibiotics, helping to avert a return to the pre-antibiotic era.

## Figures and Tables

**Table 1:** Major areas of intervention to combat antimicrobial drug resistance [3].

1.	A global public awareness campaign.
2.	Improve sanitation and hygiene to prevent the spread of infection.
3.	Reduce the unnecessary use of antimicrobials in agriculture and their dissemination in the environment.
4.	Improve the global surveillance of drug resistance and antimicrobial consumption in humans and animals.
5.	Promote new rapid diagnostics to reduce use of unnecessary antimicrobials.
6.	Promote the development and use of vaccines and alternatives.
7.	Improve the number, pay and recognition of people working in the field of infectious diseases.
8.	A global innovation fund for early stage and non-commercial research and development.
9.	Better incentives to promote investment for new drugs.

**Table 2:** Major examples of antimicrobial peptides (AMPs) in clinical trials or in development [12,13,14,15].

Antimicrobial Peptides	Indication	Phase	Company
Pexiganan (MSI-78), an analogue of magainin.	Topical cream for the treatment of diabetic foot infections and ulcers.	3	Dipexium Pharma /MacroChem/Genaera
Iseganan (IB-367), a derivative of protegrin 1.	Mouthwash for the treatment of chemotherapy induced oral mucositis.	3	Ardea Biosciences/national Cancer Institute.
	Mouthwash for the treatment of ventilator-associated pneumonia.	3	IntraBiotics Pharmaceuticals.
PAC-113 (P-113) a synthetic derivative of histatin 3 and histatin 5.	Oral gel for the treatment of candidiasis		Pacgen Biopharmaceuticals
Omiganan (MBI 226, MX-226, CSL-001), an analogue of indolicidin.	Topical cream for the treatment of skin antisepsis, prevention of catheter infections/Rosacea.	3	Mallinckrodt/Cutanea Life Sciences, Inc.
	Topical cream for the treatment of usual type vulvar intraepithelial neoplasia/moderate to severe inflammatory acne vulgaris/mild to moderate atopic dermatitis.	3	Cutanea Life Sciences, Inc.
OP-145, a derivative of LL-37.	Ear drops for the treatment of chronic bacterial middle-ear infection.	2	OctoPlus
hLF1–11, a derivative of lactoferrin.	Intravenous administration for the treatment of neutropenic stem cell transplantation patients. Prevention of bacteraemia and fungal infections.	1/2	AM Pharma.
Brilacidin, (PMX-30063), a defensin mimetic.	Intravenous administration for the treatment of acute bacterial skin and skin structure Infection caused by Gram-positive bacteria, including methicillin-resistant *Staphylococcus aureus* (MRSA).	3	Cellceutix.
	Oral rinse for the treatment of ulcerative mucositis associated with chemo/radiation therapy of cancer.	2	Cellceutix.
Arenicins, naturally occurring AMPs.	For the treatment of infections due to MDR Gram-positive bacteria.	Preclinical	Adenium Biotech
Novexatin (NP213), a synthetic AMP.	Brush on treatment for fungal infections of the toenail.	1/2	NovaBiotics
C16G2, a synthetic specifically targeted AMP.	Mouthwash for the treatment of tooth decay caused by *Streptococcus mutans*	2	C3 Jian, Inc.
Lytixar (LTX-109), a peptidomimetic.	Topical antibiotic for the treatment of nasal carriers of MRSA.	1/2	Lytix Biopharma.
	Topical cream for the treatment of infections due to Gram-positive bacteria.	2	Lytix Biopharma.

**Table 3:** AMPs with pH dependent activity.

Vertebrates	AMPs	Host Organism	Key References
Fish	Gaduscidin-1 and gaduscidin-2	*Gadus morhua*	[29,30]
Amphibians	Chensinin-1	*Rana chensinensis*	[31,32]
	Esculentin-2EM	*Glandirana emeljanovi*	This work
	Dermaseptin PD-3-7	*Pachymedusa dacnicolor*	[33]
Humans	Phagocytin		[24,25]
	Psoriasin		[34,35,36]
	β-microseminoprotein		[37]
	LL-37		[38]
	Hep-25 and hep-20		[39,40,41,42,43,44]
	Histatins		[45,46]
	Lactoferrin		[47]
	DCD-1(L)		[48,49,50]
	Kappacin A and kappacin B		[51,52]
Rabbits	Phagocytin		[24,25]
	Platelet microbiocidal proteins		[53]
	NP1 and NP2		[54,55]
Horses	Phagocytin		[24,25]
Guinea pigs	Phagocytin		[24,25]
Mice	CRAMP		[38]
Cattle	Lactoferricin B		[56,57].
**Invertebrates**	**AMPs**	**Host Organism**	**Key References**
Marine	Myticin C	*Mytilus galloprovincialis*	[58,59]
	KPS-1	*Atrina pectinate*	[60]
	Ci-PAP-A22 and Ci-MAM-A24	*Ciona intestinalis*	[61,62,63,64]
	Clavaspirin and clavanins	*Styela clava*	[65,66,67,68,69,70,71,72,73,74,75,76]
	Styelins	*Styela clava*	[68,69,77]
Terrestrial	Hebraein	*Amblyomma hebraeum*	[78]
	Amoebapores	*Entamoeba histolytica*	[79,80,81,82,83]
	Acanthaporin	*Acanthamoeba culbertsoni*	[84]
	Caenopores	*Caenorhabditis elegans*	[85,86,87,88,89]

**Table 4:** The α-helical content and lysis levels of E2EM-lin.

Lipid	pH	Lysis (%)	α-Helicity (%)
Dimyristoyl-phosphatidylserine (DMPS)	6	17	30
	8	63	49
Dimyristoyl- phosphatidylglycerol (DMPG)	6	23	51
	8	94	73
Dimyristoyl-phosphatidylcholine (DMPC)	6	52	45
	8	73	15
Dimyristoyl-phosphatidylethanolamine (DMPE)	6	60	49
	8	83	31

The levels of lysis exhibited by E2EM-lin were determined using a calcein release assay and the levels of α-helicity shown by the peptide were measured using CD spectroscopy, all as previously described [105].
